# Acceptability of escitalopram versus duloxetine in outpatients with depression who did not respond to initial second‐generation antidepressants: Study protocol for a randomized, parallel‐group, non‐inferiority trial

**DOI:** 10.1002/npr2.12078

**Published:** 2019-09-18

**Authors:** Yuma Yokoi, Atsuo Nakagawa, Naoki Yoshimura, Toshiaki A. Furukawa, Masaru Mimura, Akira Iwanami, Takayuki Abe, Kazuyuki Nakagome

**Affiliations:** ^1^ Department of Psychiatry National Center of Neurology and Psychiatry National Center Hospital Tokyo Japan; ^2^ Keio University Hospital Clinical and Translational Research Center Tokyo Japan; ^3^ Department of Health Promotion and Human Behavior and of Clinical Epidemiology Kyoto University Graduate School of Medicine/School of Public Health Kyoto Japan; ^4^ Department of Neuropsychiatry Keio University School of Medicine Tokyo Japan; ^5^ Department of Psychiatry Showa University School of Medicine Tokyo Japan; ^6^ National Center of Neurology and Psychiatry National Institute of Mental Health Tokyo Japan

**Keywords:** acceptability, antidepressants, duloxetine, escitalopram, randomized controlled trial

## Abstract

**Aim:**

The purpose of this study is to compare acceptability of two second generation antidepressants for major depressive disorder patients who have not responded to the first antidepressant for current episode. We will investigate the treatment discontinuation rate and treatment adherence as well as incidence of adverse events in order to evaluate safety.

**Methods:**

This is a two‐arm, three‐phased randomized controlled trial in which independent assessors will be blinded while treating psychiatrists and patients remain unblinded to treatment allocation. Patients will be randomized to escitalopram or duloxetine in Step 1 (8 weeks), and when entering Step 2 (8 weeks), the drug will be switched to the other if the first one is not effective at the end of Step 1. The acceptability of the allocated drugs, improvements in depression from baseline, adverse events, and attrition rates will be recorded and assessed for up to 52 weeks, including the follow‐up step.

**Results:**

It is going to be disseminated via our following reports or presentations.

**Conclusions:**

This study will provide valuable information for clinicians who encounter patients who failed to respond to their first treatment.

## Abstract 2

This is a protocol of randomized controlled trial assessing acceptability of two antidepressants after one antidepressant therapy failed.

## Graphical abstract

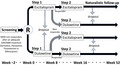

**Abbreviations**
AE: adverse event
CGI‐I: clinical global impression of improvement
CGI‐S: clinical global impression of severity
DSMC: data safety monitoring committee
DSM‐IV: Diagnostic and Statistical Manual of Mental Disorders, Fourth Edition
EC: ethics committee
ECG: electrocardiogram
EDC: electronic data capture
IRB: institutional review board
ITT: intention to treat
M.I.N.I.: Mini‐International Neuropsychiatric Interview
MDD: major depressive disorder
SAE: severe adverse event
SNRI: serotonin and norepinephrine reuptake inhibitor
SPIRIT: Standard Protocol Items: Recommendations for Interventional Trials
SSRI: selective serotonin reuptake inhibitor

## 1 BACKGROUND

Major depressive disorder (MDD) is a highly prevalent mental disorder characterized by a combination of persistent symptoms including a depressed mood, loss of interest, loss of appetite, insomnia, fatigue, difficulties with concentration, extreme guilt, and suicidal ideation.^1^ Community epidemiological studies reported that the lifetime prevalence of MDD was between 15% and 17%,^1^ while its 12‐month prevalence was between 6% and 7%.^2^ Marked impairments in social and occupational functioning are also frequently observed in patients with MDD, which was estimated to be the 11th leading cause of disability‐adjusted life years among all diseases of humankind in 2010.^3^ Furthermore, most patients with MDD will experience recurrence.^4^, ^5^ Approximately 40% of patients who recover from a major depressive episode will experience recurrence within 12 months,^6^ and this percentage increases to 85% after 15 years.^7^

Antidepressants are the most widely used treatment in daily clinical practice for this debilitating mental disorder.^8^ Evidence from numerous clinical trials has supported the efficacy of antidepressants, particularly among patients with moderate to severe MDD.^9^ The use of antidepressants has markedly increased in the last two decades since the emergence of selective serotonin reuptake inhibitors (SSRI), which are now the first‐line treatment in many countries.^8^, ^10^, ^11^ Despite these recent advances in the treatment of depression, only 50% of patients treated with antidepressants respond, and only one third achieve remission.^12^ Patients with only partial or no responses have a poorer prognosis^5^, ^13^ and reduced physical and social functions that may result in a lifelong illness.^14^ Therefore, the switching of drugs is often considered if the patient does not respond sufficiently to the first‐line treatment. Ruhe et al^15^ reported in their systematic review that the treatment response rate was 50%‐70% following a switch to another SSRI when the first‐line SSRI treatment was not effective, and switching to antidepressants with dual actions, including serotonin and norepinephrine reuptake inhibitors (SNRI), showed response rates of between 28% and 50%. Although both treatments are effective, differences in the effects of the underlying pharmacological mechanisms have not yet been elucidated.

Escitalopram is one of the antidepressants that have been categorized as SSRI, the mechanism of action of which is presumed to be linked to the potentiation of serotonergic activity in the central nervous system resulting from its inhibition of the neuronal reuptake of serotonin. Escitalopram is the S‐enantiomer of citalopram, which exhibits weak affinity for receptors other than serotonin.^16^ The efficacy of escitalopram for MDD was demonstrated in several acute 8‐week placebo‐controlled studies,^17^, ^18^, ^19^ and another 52‐week double‐blind controlled study showed that escitalopram was safe, well tolerated, and reduced the risk of relapse in long‐term treatments.^20^ Common side effects of escitalopram are nausea, headaches, dry mouth, and somnolence. In addition, a dose‐response relationship with an increased QTc interval was reported for escitalopram, albeit with a modest magnitude, in a large pharmacovigilance study.^21^

Previous findings suggest that SNRI have inherently greater efficacy than SSRI as a class.^22^, ^23^, ^24^, ^25^ Duloxetine selectively binds to norepinephrine and serotonin (5‐HT) transporters, but lacks affinity for other monoamine receptors within the central nervous system.^26^ Common side effects include nausea, headaches, dry mouth, and insomnia.^27^ The efficacy and safety of duloxetine in the acute treatment of MDD have also been established in several placebo‐controlled studies^28^, ^29^, ^30^ and one long‐term, 52‐week open‐label study.^31^

Three randomized controlled trials (RCTs)^32^, ^33^, ^34^ directly compared escitalopram and duloxetine and a meta‐analysis synthetizing the two antidepressants,^35^ and the findings obtained suggested that escitalopram has better efficacy (OR: 1.30, 95% confidence interval (95% CI): 0.88‐1.91) and a lower dropout rate (OR: 0.52, 95% CI: 0.26‐1.01) than duloxetine. However, two^33^, ^34^ of the three RCTs were conducted by the pharmaceutical company that developed escitalopram, while the other^32^ was conducted by the company that developed duloxetine. One RCT^33^ conducted by the pharmaceutical company that developed escitalopram applied an imbalanced design with flexible dosing on escitalopram and fixed dosing on duloxetine, resulting in attrition rates for escitalopram and duloxetine of 13.1% and 30.8%, respectively. In contrast, a network meta‐analysis among 12 antidepressants showed a small difference in the dropout rate between escitalopram and duloxetine (NNH = 20).^35^

These trials independently analyzed efficacy and safety; however, more general outcome measures are needed in order to assess the clinical effectiveness of antidepressants. Acceptability, namely the continuity of treatment, has become popular as a potential outcome measure of clinical effectiveness. Although limited information is currently available on acceptability using RCTs, database research^36^, ^37^ suggests that a continuous treatment with an antidepressant for more than 6 months reduces the risk of recurrence 2 years after recovery. The Vantaa Depression Study, a prospective observational study,^38^ reported that 66% of patients who succeeded with continuous treatment in the acute phase maintained remission 18 months later, whereas the other 34% did not. Moreover, adherence to the antidepressant by week 50 was reported to be 50% and the authors of the Vantaa Depression Study concluded that acceptability is the most important factor for improving the effectiveness of antidepressants.

Since prospective RCTs have not yet been conducted to investigate the acceptability and effectiveness of SSRI and SNRI, we planned this study and will compare longitudinal acceptability and effectiveness between escitalopram, the purest SSRI, and duloxetine, the most common SNRI, among patients with MDD of at least moderate severity whose responses to initial SSRI or SNRI therapy were suboptimal in a pragmatic design. This study was designed to test the hypothesis that escitalopram will be at least acceptable as and non‐inferior to duloxetine as a second‐line treatment among patients with non‐psychotic MDD who did not respond to the initial antidepressant treatment.

## 2 OBJECTIVES

The primary objective of the present study is to demonstrate the non‐inferiority of escitalopram to duloxetine in acceptability (treatment discontinuation rate) at week 52 in an RCT for patients with non‐psychotic MDD, as defined by the Diagnostic and Statistical Manual of Mental Disorders, Fourth Edition (DSM‐IV), who were identified as suboptimal responders exhibiting symptoms of at least moderate severity (scoring at least four points on the Clinical Global Impression of Severity score (CGI‐S^39^) after at least 3 weeks of treatment with second‐generation antidepressants other than escitalopram and duloxetine at a therapeutic dose.

The secondary objectives of this study are as follows: (a) to compare acceptability (treatment discontinuation rate) at week 8 and week 16 between the allocated treatment, (b) to compare acceptability (treatment discontinuation rate) during week 16 and 52 between the allocated treatment, (c) to compare the proportion of cases who terminate the trial due to the achievement of remission for more than 6 months, (d) to compare efficacy at week 8, week 16, and week 52 between the allocated treatment in terms of severity, responses, and remission rates, and (e) to compare health outcomes at week 8, week 16, and week 52 between the allocated treatment. We will also investigate the incidence of adverse events in order to evaluate safety.

## 3 METHODS/DESIGN

### 3.1 Summary of the study design

This is a randomized, flexible‐dose, parallel‐group, multi‐center trial. An 8‐week initial treatment (Step 1) is followed by a sequential 8‐week treatment (Step 2) and a naturalistic follow‐up step up to 52 weeks from the baseline. In Step 1, patients will be randomly allocated to receive escitalopram or duloxetine. In the sequential treatment in Step 2, the allocated study drug will be switched to the other study drug among non‐responders (ie, duloxetine in Step 1 and escitalopram in Step 2, and vice versa), or will be continued among responders.

### 3.2 Recruitment, screening, and baseline assessment

Recruitment is processed at participating clinical sites, which consist of two university hospitals (Keio and Showa Universities) located in central Tokyo along with their branch hospitals and clinics, and the National Center of Neurology and Psychiatry (NCNP) located in the outskirts of Tokyo, Japan.

Potential patients are referred by their treating psychiatrists, who provide brief information, during ordinal appointments, about the study using a brochure. When patients express interest in participation, a research team arranges an appointment with the patient for a detailed explanation. Not only the study design, but also its potential benefits and risks are thoroughly disclosed. Informed consent with written documents will be obtained by the investigator or a person designated by the investigator when the patient agrees to participate in the study. Patients will be screened by a treating psychiatrist in order to establish whether they meet the inclusion and exclusion criteria (described below). The M.I.N.I.^40^ (Mini‐International Neuropsychiatric Interview) will be used to confirm the MDD diagnosis and also comorbid Axis I diagnosis in order to exclude cases if other primary psychiatric disorders exist. The severity of depression will also be evaluated by PHQ‐9 41 (Patients Health Questionnaire‐9 items) and CGI‐S 39 by their treating psychiatrist. Every treating psychiatrist has been trained in the administration of semi‐structured interviews.

Demographic and clinical characteristics, including the duration of the depressive episode, will be assessed. Furthermore, an electrocardiogram (ECG) will be performed to rule out extreme QTc prolongation defined in the exclusion criteria. Standard ECG or alternative portable ECG (READ MY HEART Plus®) (http://www.jhf.or.jp/ecg/type.html) will be used to evaluate QTc. Blood drawing will also be conducted.

Baseline assessments will be scheduled within no longer than 2 weeks from screening for those who meet inclusion and exclusion criteria. At the baseline, the severity of depression will be assessed by QIDS‐SR_16_ 42 (16 items Quick Inventory of Depressive Symptomatology—Self‐Reported) and CGI‐S. Quality of life will be assessed by EQ‐5D^43^ (European Quality of Life Questionnaire‐5 Dimension). Central raters will evaluate depression severity by PHQ‐9 and the level of functional impairment by the Sheehan Disability Scale (SDS)^44^ via telephone.

Randomization will be conducted using the central computerized registration system and the subject will be allocated to receive one of the study medications (escitalopram or duloxetine), which is conveyed to the treating psychiatrist by a clinical research coordinator, and will be tapered from their current antidepressant (switched by the cross‐titration and tapering method) by week 4 in the Step 1 treatment. Treatment allocation is blinded to central raters throughout this study.

### 3.3 Patients

#### 3.3.1 Inclusion criteria

1. Fulfill criteria for MDD, as defined by the DSM‐IV criteria for single or recurrent MDD without psychotic features, as evaluated by a clinical assessment by the treating psychiatrist and confirmed by the M.I.N.I.
2. Aged 20‐65 years at screening.
3. Patients who have been treated with a therapeutic dose of SSRI (sertraline, paroxetine, or fluvoxamine), SNRI (milnacipran) for at least 3 weeks.
4. Depressive symptoms of at least moderate severity based on CGI‐S score ≥ 4.
5. MDD is the primary diagnosis, and the treating psychiatrist has judged the study medication (ie, escitalopram or duloxetine) to be appropriate for prescription.
6. Competent and able to understand the meaning of the observation, evaluation, and clinical examination in the judgment of the treating psychiatrist.
7. Competent and able to give their own informed consent.
8. Available on the telephone for assessments.

#### 3.3.2 Exclusion criteria

1. Did not respond to two or more adequate antidepressants (each for at least 4 weeks at a therapeutic dose) during a current depressive episode judged by the treating psychiatrist.
2. Comorbid psychiatric condition (DSM‐IV axis I) other than MDD that is regarded as the primary diagnosis within 1 year of screening.
3. History of bipolar disorder, schizophrenia, or other psychotic disorders at screening decided by the treating psychiatrist.
4. History of substance abuse/dependence within 1 year of screening, except caffeine, and nicotine.
5. Have an Axis II disorder that, judged by the treating psychiatrist, may interfere with compliance with the study protocol.
6. Did not respond to escitalopram or duloxetine at the maximum dose for at least 4 weeks during the previous depressive episode.
7. Women who are currently pregnant or breastfeeding.
8. Patients who are judged by the treating psychiatrist to be at serious risk of harm to themselves or others.
9. Patients who are judged by the treating psychiatrist to have serious and/or unstable illness, such as diseases in the liver, kidneys, respiratory system, hematological system, endocrine system, or central nervous system, including traumatic brain injury.
10. Have a serious or unstable cardiovascular illness (including severe arrhythmia with bradycardia, prescribed drugs known to cause QTc prolongation, congestive heart failure, and hypokalemia) or a clinically significant ECG abnormality (male: QTc > 450 ms, female: QTc > 470 ms).
11. Ongoing treatment with monoamine oxidase inhibitors within 2 weeks before the screening.
12. Have uncontrolled closed‐angle glaucoma.
13. Ongoing treatment with pimozide (Orap®).
14. Patients who are judged by the treating psychiatrist to be inappropriate to participate in the study.

### 3.4 The trial schedule

**Figure 1:**
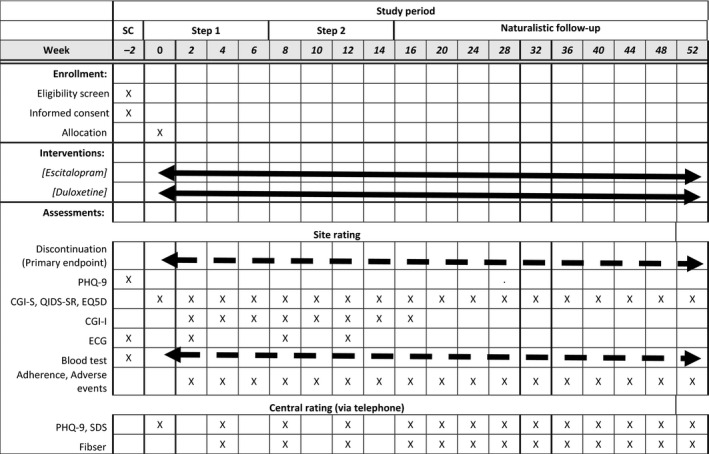
Schedule of enrollment, interventions, and assessments. As long as possible. As needed. CGI‐I, clinical global impression of improvement; ECG, electrocardiogram; EQ5D, European Quality of Life Questionnaire – 5 Dimension; PHQ‐9, Patient Healthcare Questionnaire – 9 items; QIDS‐SR, Quick Inventory of Depressive Symptomatology—Self‐Reported; SC, screening; SDS, Sheehan Disability Scale

**Figure 2:**
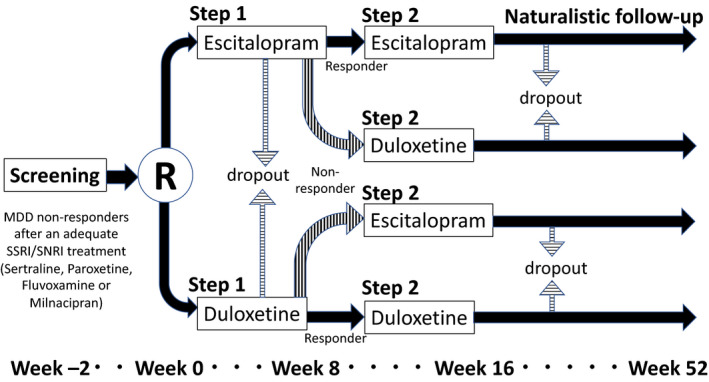
The scheme for trial schedule. MDD, major depressive disorder; R, randomization; SNRI, serotonin and norepinephrine reuptake inhibitor; SSRI, selective serotonin reuptake inhibitor

The schedules for observations and examinations are shown in Figure 1 in accordance with SPIRIT^45^ diagram (SPIRIT checklist of this protocol is on Appendix [Link]) and the scheme of trial schedule in Figure 2. The trial consists of three steps: Step 1 for the initial allocated treatment, Step 2 for the continuous or switched treatment, and the naturalistic follow‐up step until week 52.

#### 3.4.1 Step 1 treatment

During the Step 1 treatment, the protocol strongly encourages all patients to receive an adequate dose of the allocated medication (escitalopram 10‐20 mg/d starting from 10 mg/d, or duloxetine 40‐60 mg/d starting from 20 mg/d) for 8 weeks using a flexible‐dose schedule to maximize the chance of remission.

The recommended treatment visits will be bi‐weekly, and optional visits are allowed between bi‐weekly visits if needed. Patient compliance with the study antidepressant will be assessed at each bi‐weekly visit. The decision to proceed to the next step depends on the clinical judgment of the treating psychiatrist regarding the benefit of the current treatment based on clinical assessment information (CGI‐S and clinical global impression of improvement (CGI‐I)) obtained during the study.

#### 3.4.2 Treatment evaluation and possible switching

Patients who fulfill either of the switching criteria described below will be discontinued from the currently allocated medication and switched to the other study medication for the subsequent 8‐week treatment using the cross‐titration and tapering method. On the other hand, those who do not fulfill the switching criteria (successful treatment) will be encouraged to maintain the currently allocated medication.

1. Unsuccessful treatment: Defined as patients with (a) CGI‐S score ≥ 4 (ie, moderately ill) and (b) CGI‐I^39^ score ≥ 4 (ie, no change or worse) evaluated at week 8 (end of the Step 1 treatment).
2. Discontinuation due to “intolerance,” “ineffectiveness,” or “worsening” of the allocated medication at any timepoint.

#### 3.4.3 Step 2 treatment and naturalistic follow‐up

The protocol strongly encourages patients to continue Step 1 medication if patients do not meet the switching criteria and to alter Step 1 medication if they do. They receive another 8‐week treatment using a flexible‐dose schedule to maximize the chance of remission, except for those with clear intolerance or significant clinical worsening based on CGI‐I score ≥ 6 at two consecutive visits. The recommended schedule is bi‐weekly appointments as well as the Step 1 treatment, and optional visits are allowed in accordance with clinical needs.

After week 16, all patients will be followed up to week 52 in order to assess adherence, efficacy, adverse events, and natural courses. There will be no prohibited treatment during the follow‐up, and patients will be encouraged to visit every 4 weeks even if antidepressants are discontinued.

#### 3.4.4 Discontinuation during Step 1 and Step 2

Discontinuation from the allocated treatment (in Step 1 and Step 2) will be decided as follows:

1. The treating psychiatrist's judgment that disadvantages from adverse effects outweigh benefits from continuing the allocated treatment.
2. Patient wishes for withdrawal from the allocated drug.
3. Significant clinical worsening based on CGI‐I score ≥ 6 (ie, much worse or very much worse) at two consecutive visits.
4. Urgent risk of suicide, self‐injury, or harming others.
5. Getting pregnant.
6. Occurrence of severe or unstable comorbidities including cardiovascular, hepatic, renal, respiratory, hematological, endocrinal, and neurological diseases.
7. Occurrence of a clinically significant abnormality in ECG (QTc > 450 ms in men, >470 ms in women).
8. Clinical judgment by the treating psychiatrist that the continuation of the allocated treatment is not possible, inappropriate, or unsuitable for other reasons.
9. Observation of major protocol deviations.

When the allocated treatment is halted, the treating psychiatrist will explain this to the subject. In the case of adverse effects, appropriate observations, examinations, and procedures are implemented by the treating psychiatrist. Following the discontinuation of the allocated treatment in Step 1 and Step 2, patients are still followed up until week 52 unless they withdraw their consent. Similarly, even in the case of major protocol deviations, patients will be followed up until week 52, except for the withdrawal of consent by the subject or discontinuation decided by the treating psychiatrists.

#### 3.4.5 Deviation from the protocol

The following cases will be potential deviations from the protocol. Patients with these deviations will not be automatically excluded from the study, they will receive protocol assessments.

1. Failure to taper off prior antidepressants within 4 weeks from study initiation.
2. Concomitant use of antidepressants, antipsychotics, sulpiride, mood stabilizers (lithium carbonate, carbamazepine, valproic acid, lamotrigine, and other antiepileptics), and St. John's Wort during Step 1 and Step 2.
3. Structured psychological interventions, such as cognitive behavioral therapy, during Step 1 and Step 2.
4. Electroconvulsive therapy or repetitive transcranial magnetic stimulation during Step 1 and Step 2.
5. Absence of two consecutive visits, which may result in the lack of study evaluations and withdrawal from the study.

### 3.5 Outcome measures

#### 3.5.1 Primary outcome measures

The primary endpoint of the present study is the rate of discontinuation for any reason at week 52. The discontinuation of study medication will be further divided into seven subgroups based on the reason for discontinuation ascertained by the treating psychiatrist at the time of the subject's discontinuation and will be confirmed by the Data Safety Monitoring Committee (DSMC).

1. Discontinuation due to intolerance is deemed by the treating psychiatrist while its validity needs to be evaluated by the scores of FIBSER^46^ (Frequency, Intensity, and Burden of Side Effects Rating) and EQ5D.
2. Discontinuation due to the lack of efficacy is applied when initiated by patients or their related persons claiming that the allocated drug is not effective and they want to stop using it.
3. Discontinuation due to significant deterioration is defined as CGI‐I score ≥ 6 (worse or markedly worse) in two consecutive visits. Treating psychiatrists must stop using the allocated drug.
4. Discontinuation due to consent withdrawal is applied when a written withdrawal form is submitted by the subject. Reasons for withdrawal will be further analyzed and may be reclassified to other reasons for discontinuation.
5. Discontinuation due to other safety issues, such as gestation, accidents, or admission to hospital with a physical illness, is deemed by the treating psychiatrist or patients.
6. Discontinuation due to a loss to the follow‐up for inevitable reasons such as moving.
7. Discontinuation due to other reasons.

#### 3.5.2 Secondary outcome measures

##### Discontinuation

1. The rate of discontinuation of study medication for any reason during Step 1 and Step 2.
2. The rate of discontinuation of study medication for any reason during periods outside Step 1 and Step 2.
3. Occurrence of treatment cessation because of recovery from illness (CGI‐S score = 1 for more than 6 months and in case the patient and the treatment psychiatrist agreed to stop medication).

##### Efficacy

1. Change in the severity of depression as measured by PHQ‐9 (central rating), CGI‐S, and QIDS‐SR_16_ (on site).
2. The proportion of responders. Responders will be defined as those who score a 50% or greater reduction from the baseline PHQ‐9 score.
3. The proportion of remission. Remission will be defined as the PHQ‐9 score ≤ 4.

##### Health outcomes

1. Change in the level of quality of life as measured by the EQ‐5D (on site).
2. Change in the level of functional impairment as measured by the SDS^44^ (central rating).

##### Safety

1. Change in the level of the global burden of side effects as measured by the FIBSER Scale (central rating).
2. Spontaneously reported Adverse Events (AEs) report and Serious Adverse Events (SAEs) report (on site).
3. The onset of manic/hypomanic/mixed episodes evaluated by a clinical assessment and confirmed by the M.I.N.I. (on site).
4. ECGs (QTc) and laboratory analyses (CBC, TP, Alb, T‐Bil, AST, ALT, GGTP, BUN, Cre, T‐Chol, TG, Na, K, CL, and glucose).

##### AEs

Adverse events are defined as any undesirable experience in patients during the use of medication. Regardless of the relationship and causation to the medication, all AEs are monitored and recorded throughout the study by clinical evaluations and observations, the results of any clinical data, and clinical complaints. Serious Adverse Events are defined as any untoward medical occurrence that may happen at any dose:

1. Results in death.
2. Is life‐threatening.
3. Requires inpatient hospitalization or causes the prolongation of existing hospitalization.
4. Results in persistent or significant disability/incapacity.
5. Is a congenital anomaly/birth defect, or
6. Requires interventions to prevent permanent impairment damage.

However, hospitalization not related to the exacerbation of the current mental illness, such as hospitalization for the coordination of social circumstances, judged by the treating psychiatrist, is not regarded as SAE. Treating psychiatrists record the name (defined in Medical Dictionary for Regulatory Activities, MedDRA), occurrence date, seriousness, grade, any intervention for AE (the name of the intervention if any), outcome, and relationship to the study medication into the electronic clinical research form (eCRF). If the symptoms already existed at the baseline and no exacerbation occurred during the study, they will not be treated as AEs.

Adverse events are graded into three categories in the present study:

- Mild: asymptomatic or mild symptoms; clinical or diagnostic observations only; no intervention indicated; no effects on usual daily activities.
- Moderate: minimal, local, or non‐invasive intervention indicated; limiting age‐appropriate instrumental activities of daily living (ADL); some effects on usual daily activities.
- Severe: medically significant, life‐threatening, or relating to death; hospitalization or the prolongation of hospitalization indicated; disabling; limiting self‐care ADL; significant effects on usual daily activities.

Causality between AEs and the medication is judged by the treating psychiatrist as one of the following:

1. No causality: There is no time relationship between the occurrence and study drug, and it is supposed to be from the underlying disease, comorbidities, accidentally, medication other than the study drugs, or comorbid therapies.
2. Possible: There is some time relationship, and it may not be from the underlying disease, comorbidities, accidentally, medication other than the study drugs, or comorbid therapies. Considering toxicity or the pharmacological action of the study drug, the relationship of the study drug cannot be excluded.
3. Probable: There is some time relationship and it cannot be from the underlying disease, comorbidities, accidentally, medication other than the study drugs, or comorbid therapies. The toxicity or pharmacological action of the study drug clearly explains the relationship.
4. Unclear: None of the above.

Outcomes are judged by the treating psychiatrist as follows:

1. Full recovery: disappearance of AE and the exact status as that before AE.
2. Partial recovery: improvement in the degree of AE despite its existence.
3. No recovery: no improvement or deterioration in the degree of AE.
4. Full recovery with sequelae: disappearance of AE with some sequelae.
5. Death: death.
6. Unknown: unable to identify (such as due to a no‐show).

Procedures and interventions for AEs are as follows:

1. The treating psychiatrist will monitor and take responsibility for the patient's safety.
2. If they judge that any intervention or treatment is necessary, the treating psychiatrist will explain to the subject the details of what he/she will do and the expected effects and risks.
3. In addition to taking appropriate medical interventions, the treating psychiatrist will follow up the subject for a reasonable period of time, record the AE name, date of occurrence, seriousness, degree, degree of treatment, presence or absence of treatment, outcome, and causal relationship.
4. If SAEs occur, the treating psychiatrist and principle investigator will report their details to the Institutional Review Boards (IRBs)/Ethical Committees (ECs) and DSMC immediately to follow their decision as to whether the study may be ethically continued and if the protocol and/or informed consent form need to be amended following SAEs.
5. The contents of medical records will be reported to the study office by the treating psychiatrist or clinical research coordinator via the eCRF.
6. At least annually, the principle investigator summarizes the status of the research, the occurrence of adverse events, and self‐assessments, and the clinical trial review committee or ethics review committee of each facility evaluates the research implementation status report.

##### Methods of data collection and management

In the present study, all data, anonymized removing all obvious identifiers such as name, address, will be entered electronically via online clinical data capture, named the CR system. Original study forms will be filed and stored in locked cabinets at each participating site. They will be adequately stored for 5 years after completion of this study. Access to these forms will be properly restricted. Access to the data entered will be logged and restricted by identification codes and passwords. Data to be collected at each visit will be reviewed timely in order to check for inconsistencies by the data manager at the NCNP. The study sites are responsible for making appropriate corrections to the original paper forms whenever any item will be changed. Changes in electronic data will be preserved and traceable via electronic data capture and audit trails.

Data collection forms and required information will be discussed, and coordinators will be trained on how to code symptoms, events, and diseases using MedDRA software. They will also learn to enter data, respond to queries regarding data discrepancies, obtain general information, and arrange it into research quality data during training.

### 3.6 Statistical analysis

#### 3.6.1 Sample size calculation

The sample size needed will be 242 (n = 121 per group) in order to demonstrate the non‐inferiority of escitalopram to duloxetine with respect to the proportion of patients who discontinue the study at week 52. The sample size is based on the findings of the MANGA meta‐analysis,^35^ which estimated the between‐group difference in the attrition rate at week 52 to be 8%, and the attrition rate of escitalopram at week 52 was inferred to be 49% according to a previous study.^20^ Using a non‐inferiority margin of 10%, 242 patients need to be enrolled in order to achieve a statistical power of .8 with one‐sided alpha = .05.^35^

#### 3.6.2 Statistical analysis plan

Details on statistical analyses are specified in the statistical analysis plan (SAP) prepared and finalized before the analysis, although the basic policy is to compare groups for the items described below. The significance level in testing the primary non‐inferiority hypothesis is one‐sided .05 and that for all other secondary endpoints is one‐sided .05.

#### 3.6.3 Primary analyses

The primary analysis population in this study is ITT (intention to treat). All patients enrolled in this study need to be included in the analysis, except those who did not take the study medication.

Acceptability—Our primary endpoint is attrition and withdrawal rates from the allocated treatment for any reason at week 52. The non‐inferiority of escitalopram to duloxetine will be tested using the one‐sided 95% CI for the between‐group difference in the discontinuation rate (escitalopram − duloxetine). The CI is estimated with the normal approximation. If the upper limit of the one‐sided 95% CI is less than the non‐inferiority margin (Δ = 10%), escitalopram is considered to be non‐inferior to duloxetine in the primary endpoint.

#### 3.6.4 Secondary analyses

##### Interim analyses

We will conduct interim analyses regarding safety following 100 registered patients. Data Safety Monitoring Committee will review the data to judge whether it is unethical to implement this study.

##### Analyses for secondary outcomes

Regarding repeated measurements of continuous endpoints in Step 1, a mixed‐effects model for repeated measurements (MMRM) with an unstructured covariance matrix will be used to compare mean changes from the baseline between two groups. Degrees of freedom for errors will be adjusted with the Kenward‐Roger method. The 95% CI for the proportion in each binary endpoint will be estimated using the Clopper‐Pearson method, and proportions will be compared with the chi‐squared test. In analyses of Step 2 data, the proportion of patients who switched the study medication will be summarized, and data will be summarized for the randomized group and for each pattern of the switched treatment.

#### 3.6.5 Monitoring

We have an independent DSMC. Data Safety Monitoring Committee assess progress, safety data, and critical efficacy endpoints at intervals not only to monitor trial safety, but also participant recruitment, protocol compliance, and data quality in order to ensure that clinical equipoise is maintained during the trial and also to recommend to the sponsor whether to continue, modify, or stop the trial. Data Safety Monitoring Committee is composed of three clinicians with expertise in clinical trials and a biostatistician is added only in the case of interim analyses. Audits will be conducted twice during the study, the first following the start of the study and the second after the first case enrollment, in order to evaluate the study processes and quality control by individuals who are independent of the study.

## 4 DISCUSSION

The ACCEPT study is a pragmatic multi‐center, randomized, parallel‐group, controlled trial, which compares the acceptability of escitalopram and duloxetine as a second‐line treatment. It is generally difficult for a clinician to select the most appropriate drug for a patient because evidence for efficacy and tolerability often suggest different approaches. Acceptability, namely the continuity of treatment is a more general outcome measure and, thus, may provide clearer indications to a physician in drug selection. A relationship between acceptability and clinical effectiveness has been suggested in observational studies, whereas no RCTs have been conducted. Additionally, acceptability is the most applicable outcome for busy clinicians encountering tens or hundreds of patients each day in Japan. Therefore, this study will provide important information when physicians fail to treat MDD patients with the first‐line antidepressant treatment.

The biggest issue in implementing this study is to reduce the number of patients lost to the follow‐up, which may cause bias in the interpretation of study results. Any reason for protocol deviations or non‐adherence to assigned treatments need to be fully recorded and analyzed in order to minimize or prevent potential biases. The status of patients will be monitored real‐time and followed up as long as possible up to week 52 unless patients withdraw consent.

The reinforcement of recruitment in real‐world clinics is another important issue that needs to be solved. More than 50% of the study sites are private clinics at which physicians see more than 50 patients per day. In order to reduce the burden on clinicians and save time enrolling and seeing patients, the severity of depression and drug safety evaluations via the telephone are introduced. Independent evaluations also contribute to the blind assessment of some secondary outcomes and validation of the clinical judgment by the treating psychiatrist. Clinical global impression and other scales to be assessed by the treating psychiatrist may be promptly (within an additional 1‐2 minutes) executed during busy appointments otherwise enrollment in real‐world clinics will be impossible.

This protocol considers the above discussed and other undescribed issues and takes appropriate measures to enhance and enable the large number of patients enrolled.

## CONFLICT OF INTEREST

YY has received speaker's honoraria from Eisai and research funding from Chugai Pharmaceutical, Eisai, Eli Lilly Japan, Biogen Japan, Meiji Seika Pharma, and Sumitomo Dainippon Pharma. AN has received lecture fees from Meiji, Pfizer, Eli Lilly, Otsuka Pharmaceutical, Janssen Pharmaceutical, Takeda, Mitsubishi Tanabe Pharma, Mochida Pharmaceutical, Sumitomo Dainippon Pharma, and NTT Docomo, participated in an advisory board for Takeda, and received research funding from Johnson & Johnson. TAF has received lecture fees from Janssen Pharmaceutical, Meiji, Mitsubishi Tanabe Pharma, MSD, and Pfizer. He has received research support from Mitsubishi Tanabe Pharma and Mochida. MM has received grants and/or speaker's honoraria from Asahi Kasei Pharma, Astellas Pharmaceutical, Daiichi Sankyo, Sumitomo Dainippon‐Pharma, Eisai, Eli Lilly, Fuji Film RI Pharma, Janssen Pharmaceutical, Kracie, Meiji‐Seika Pharma, Mochida Pharmaceutical, MSD, Novartis Pharma, Ono Yakuhin, Otsuka Pharmaceutical, Pfizer, Shionogi, Takeda, Mitsubishi Tanabe Pharma, and Yoshitomi. AI has received grants and/or lecture fees from Astellas Pharmaceutical, Daiichi Sankyo, Sumitomo Dainippon Pharma, Eisai, Eli Lilly, Janssen Pharmaceutical, Meiji‐Seika Pharma, Mochida Pharmaceutical, MSD, Otsuka Pharmaceutical, Pfizer, Shionogi, Takeda, Mitsubishi Tanabe Pharma, and Yoshitomi. TA has received lecture fees from Nippon Boehringer Ingelheim, Johnson & Johnson, JCR Pharmaceutical, Reprocell, and Chugai Pharmaceutical. KN has received lecture fees from Kyowa Hakko Kirin, Otsuka Pharmaceutical, Sumitomo Dainippon Pharma, Eli Lilly, Shionogi, Meiji Seika Pharma, Pfizer, MSD, GlaxoSmithKline, Janssen Pharmaceutical, Mochida Pharmaceutical, and Yoshitomi, and participated in an advisory board for Otsuka Pharmaceutical, Nippon Boehringer Ingelheim, Takeda, Toyama Chemical, Sumitomo Dainippon Pharma, Meiji Seika Pharma, Pfizer, Janssen Pharmaceutical, and Mochida Pharmaceutical, and received research funding from Mitsubishi Tanabe Pharma, Asahi Kasei Pharma, Astellas Pharma, Mochida Pharmaceutical, Meiji Seika Pharma, Otsuka Pharmaceutical, Nippon Boehringer Ingelheim, and Sumitomo Dainippon Pharma.

## AUTHOR CONTRIBUTIONS

TF, MM, AI, and KN conceived of and designed the study. All authors contributed to forming the protocol. TA created the statistical analysis plan. YY and AN performed the literature search and drafted the manuscript. NY, TF, MM, AI, and KN critically reviewed the manuscript and contributed to the revision of the manuscript. All authors confirmed and approved the final manuscript.

## ETHICAL APPROVAL

The protocol Version 2.4 was presented to an IRB for approval (National Center of Neurology and Psychiatry IRB, MD‐KN01). Following an initial review and approval (16 October, 2013), the local institutional IRBs/ECs will review the protocol. IRBs/ECs will review the protocol at least annually. The principal investigator will submit safety and progress reports to the IRBs/ECs at least annually and investigators in each site will submit reports within 3 months of study termination or completion at the local site. These reports will include the total number of patients enrolled, severe and non‐severe adverse events, and summaries of each data safety and monitoring board review of safety and/or efficacy. At the occurrence of SAEs, the principle investigator will immediately report the details of the incidence to the NCNP‐IRB and DSMC. All patients will give written informed consent (Appendix [Link]) prior to the baseline assessment. When necessary, DSMC plays a role to recommend to the sponsor whether to modify the trial protocol taking into consideration such factors like the safety, recruitment progress, and protocol compliance. In this case, the principal investigator may apply for IRBs/ECs to review the modified protocol after discussing the matter with other investigators.

## STUDY STATUS

The study was accomplished in March 2018.

## DISSEMINATION POLICY

The results are planned to be published and/or presented for public, especially for healthcare professionals.

## Data Availability

The datasets generated and/or analyzed during the present study are not publicly available due to individual privacy issues, but are available from the corresponding author upon reasonable request.
