# Supplementary material for: Acceptability of escitalopram versus duloxetine in outpatients with depression who did not respond to initial second‐generation antidepressants: Study protocol for a randomized, parallel‐group, non‐inferiority trial
Source: Neuropsychopharmacol Rep. 2019 Sep 18;39(4):262–72. doi: 10.1002/npr2.12078 (PMC7292285; doi:10.1002/npr2.12078)
Supplement: Supplementary file 1 [file NPR2-39-262-s001.docx]

**Appendix S1**

Please read well

**Informed consent form on** **long-term acceptability study** **of** **novel antidepressants for patients with major depressive disorder**

Please read carefully before deciding

whether to participate in this research.

Please feel free to contact us

if you have any questions or concerns.

**National Center of Neurology and Psychiatry**

First Edition

Creation Date: October 16^th^, 2013

1. Purpose of research

Your illness (depression) is a sickness in which you feel badly depressed for some reason and lose interest in things. In the course of this illness, you take everything pessimistically, cannot feel joy or fun, do not feel motivated to do anything, and, as a consequence, may experience difficulties in your everyday life. Moreover, you suffer from strong mental distress, leading to a psychosomatic disorder. It has been estimated that 1 out of every 15 individuals in Japan will develop depression.

In this trial, patients showing insufficient responses to selective serotonin reuptake inhibitors (SSRI) or serotonin and norepinephrine reuptake inhibitors (SNRI), which are frequently used to treat depression in clinical settings (sertraline, paroxetine, fluvoxamine, or milnacipran), are administered escitalopram or duloxetine and followed up for 52 weeks in order to compare the continuity of the treatment of each drug as well as improvements in depressive symptoms, everyday life, and the occurrence of side effects. We also investigate whether duloxetine is effective for patients who do not respond to escitalopram, and vice versa, by switching the drugs in patients who show no effects.

1. Voluntariness of research participation and the right to withdraw

Whether you agree to participate in this research is your free choice. Even if you do not agree, you will never receive disadvantageous treatment. Even after you agree to participate in this study, you may withdraw your consent freely at any time. Even if you do not participate in the research until its completion, you will never receive disadvantageous treatment.

However, please note that if you decide not to participate in the study after taking the study medicine, you may have to undergo a medical examination in order to assess your condition. Furthermore, even if you decide to cancel participation in the midst of this research, we will use the data obtained until consent was canceled unless requested. Even in that case, your privacy will be preserved.

If new and important information about the study drugs is obtained during the research participation period, we will reconfirm your intention to continue to participate in the study.

3. Research method

1) Criteria for participation

⮚ Those who are eligible to participate

1. Fulfill the criteria for major depressive disorder (MDD), as defined by the DSM-IV criteria for single or recurrent MDD without psychotic features, as evaluated in a clinical assessment by the treating psychiatrist and confirmed by the M.I.N.I..
2. Aged 20-65 years at screening.
3. Patients who have been treated with a therapeutic dose of SSRI (sertraline, paroxetine, or fluvoxamine), SNRI (milnacipran), or noradrenergic and specific serotonergic antidepressants (mirtazapine) for at least 3 weeks.
4. Depressive symptoms of at least moderate severity based on CGI-S score ≥ 4.
5. MDD is the primary diagnosis, and the treating psychiatrist has judged the study medication (i.e., escitalopram or duloxetine) to be appropriate for prescription.
6. Competent and able to understand the meaning of the observation, evaluation, and clinical examination in the judgment of the treating psychiatrist.
7. Competent and able to give their own informed consent.
8. Available on the telephone for assessments.

⮚ Those who are ineligible to participate

1. Did not respond to two or more adequate antidepressants (each for at least 4 weeks at a therapeutic dose) during a current depressive episode judged by the treating psychiatrist.
2. Comorbid psychiatric condition (DSM-IV axis I) other than MDD that is regarded as the primary diagnosis within one year of screening.
3. History of bipolar disorder, schizophrenia, or other psychotic disorders at screening decided by the treating psychiatrist.
4. History of substance abuse/dependence within one year of screening, except caffeine and nicotine.
5. Have an Axis II disorder that, judged by the treating psychiatrist, may interfere with compliance with the study protocol.
6. Did not respond to escitalopram or duloxetine at the maximum dose for at least 4 weeks during a previous depressive episode.
7. Women who are currently pregnant or breastfeeding.
8. Patients who are judged by the treating psychiatrist to be at serious risk of harm to themselves or others.
9. Patients who are judged by the treating psychiatrist to have a serious and/or unstable illness, such as diseases of the liver, kidneys, respiratory system, hematological system, endocrine system, or central nervous system, including traumatic brain injury.
10. Have a serious or unstable cardiovascular illness (including severe arrhythmia with bradycardia, prescribed drugs known to cause QTc prolongation, congestive heart failure, and hypokalemia) or a clinically significant ECG abnormality (male: QTc>450 ms, female: QTc>470 ms).
11. Ongoing treatment with monoamine oxidase inhibitors within 2 weeks before screening.
12. Have uncontrolled closed-angle glaucoma.
13. Ongoing treatment with pimozide (Orap®).
14. Patients who are judged by the treating psychiatrist to be inappropriate to participate in the study.

There are more detailed criteria other than those listed above. We will also conduct blood tests as necessary in order to establish whether you meet the conditions for participation in the study. Depending on the results of this examination, you may not be able to participate in this research. Please contact your doctor or clinical research coordinator for more details.

1

|  | **STUDY PERIOD** | | | | | | | | | | | | | | | | | | | | | | | |
| --- | --- | --- | --- | --- | --- | --- | --- | --- | --- | --- | --- | --- | --- | --- | --- | --- | --- | --- | --- | --- | --- | --- | --- | --- |
|  | **SC** | **Step 1** | | | | | **Step 2** | | | | | **Naturalistic follow-up** | | | | | | | | | | | |  |
| **WEEK** | ***-2*** | | **0** | ***2*** | ***4*** | ***6*** | | ***8*** | ***10*** | ***12*** | ***14*** | | ***16*** | ***20*** | ***24*** | ***28*** | ***32*** | ***36*** | ***40*** | ***44*** | ***48*** | | ***52*** | |
| **ENROLLMENT:** |  | |  |  |  |  | |  |  |  |  | |  |  |  |  |  |  |  |  |  | |  | |
| Eligibility screen | X | |  |  |  |  | |  |  |  |  | |  |  |  |  |  |  |  |  |  | |  | |
| Informed consent | X | |  |  |  |  | |  |  |  |  | |  |  |  |  |  |  |  |  |  | |  | |
| Allocation |  | | X |  |  |  | |  |  |  |  | |  |  |  |  |  |  |  |  |  | |  | |
| **INTERVENTIONS:** |  | |  |  |  |  | |  |  |  |  | |  |  |  |  |  |  |  |  |  | |  | |
| *[Escitalopram]* |  | |  |  |  |  | |  |  |  |  | |  |  |  |  |  |  |  |  |  | |  | |
| *[Duloxetine]* |  | |  |  |  |  | |  |  |  |  | |  |  |  |  |  |  |  |  |  | |  | |
| **ASSESSMENTS:** |  | |  |  |  |  | |  |  |  |  | |  |  |  |  |  |  |  |  |  | |  | |
| **Site Rating** | | | | | | | | | | | | | | | | | | | | | |  |  |  |
| Discontinuation  (Primary endpoint) |  | |  |  |  |  | |  |  |  |  | |  |  |  |  |  |  |  |  |  | |  | |
| PHQ-9 | X | |  |  |  |  | |  |  |  |  | |  |  |  | . |  |  |  |  |  | |  | |
| CGI-S, QIDS, EQ5D |  | | X | X | X | X | | X | X | X | X | | X | X | X | X | X | X | X | X | X | | X | |
| CGI-I |  | |  | X | X | X | | X | X | X | X | | X |  |  |  |  |  |  |  |  | |  | |
| ECG | X | |  | X |  |  | | X |  | X |  | |  |  |  |  |  |  |  |  |  | |  | |
| Blood test | X | |  |  |  |  | |  |  |  |  | |  |  |  |  |  |  |  |  |  | |  | |
| Adherence, Adverse events |  | |  | X | X | X | | X | X | X | X | | X | X | X | X | X | X | X | X | X | | X | |
| **Central Rating (via telephone)** | | | | | | | | | | | | | | | | | | | | | |  |  |  |
| PHQ-9, SDS |  | | X |  | X |  | | X |  | X |  | | X | X | X | X | X | X | X | X | X | | X | |
| FIBSER |  | |  |  | X |  | | X |  | X |  | | X | X | X | X | X | X | X | X | X | | X | |

As long as possible

As needed

1

2) Examination/examination schedule

 R: randomization, MDD: major depressive disorder, SSRI: selective serotonin reuptake inhibitor, SNRI: serotonin and norepinephrine reuptake inhibitor.

**

*

* If it becomes difficult to continue the treatment during Step 1, it will be switched to Step 2 even if 8 weeks has not been reached at that time.

** If at least one of the following criteria is met, medication will be changed to the other study medicine. If it does not, the study medicine will not be changed.

1. Treatment ineffective: CGI-S is 4 or more and CGI-I is 4 or more

2. A patient’s intention to change medication due to poor tolerability, inadequate effects of the study medicine, or deterioration defined as CGI-I ≧ 6 on two consecutive evaluation days.

⮚ Medication

· In Step 1, you will start taking a drug, either escitalopram or duloxetine, which has been randomly assigned to you.

· The treating psychiatrist will prescribe the drug following the indication noted in the package insert based on clinical judgment.

·The study medicine selection criteria before Step 2 are as follows.

If the treating psychiatrist judges that at least one of the following criteria is met, medication will be changed to the other study medicine. If it is not, the study medicine will not be changed.

1. Treatment ineffective

2. A patient’s intention to change medication due to poor tolerability, inadequate effects of the study medicine, or deterioration.

⮚ Blood sampling

During the study, blood tests are performed as needed, including biochemical examinations (total protein, albumin, total bilirubin, GOT/GPT, γ -GTP, urea nitrogen, creatinine, total cholesterol, neutral fat, sodium, potassium, chloride, and glucose) and blood counts (red blood cell, white blood cell, hemoglobin, hematocrit, and platelet). The amount of blood drawn is 10 ml for each test.

3) Evaluation of symptoms

[Evaluation by telephone]

⚫          Depression severity (PHQ - 9: Patients Health Questionnaire - 9 items)

⚫          Functional impairment (SDS: Sheehan Disability Scale)

⚫          Global burden of side effects (FIBSER:)

0, 4, 8, 12, 16, 20, 24, 28, 32, 36, 40, 44, 48, 52 weeks,

※ Central raters who are blind to the drug assignment perform the evaluation via telephone.

[Evaluation on site]

⚫          Depression severity (QIDS - SR 16: 16 items Quick Inventory of Depressive Symptomatology Self - Reported)

⚫          Quality of life (EQ - 5 D: European Quality of Life Questionnaire - 5 Dimensions)

0, 2, 4, 6, 8, 10, 12, 14, 16, 20, 24, 28, 32, 36, 40, 44, 48, 52 weeks, or when the patient has dropped out

※ We ask for your cooperation with self-evaluations.

4) Prohibited concomitant drugs and foods

In order to investigate the effectiveness and safety of the study medicine, there are drugs that are prohibited to use concomitantly during Step 1 and Step 2. Furthermore, during Step 1 and Step 2, please do not markedly change your intake of caffeine or nicotine.

Please tell your doctor or clinical research coordinator of all the medications you are currently taking. In addition, if you plan to start taking medicine or if you bought new medicine from a pharmacy, please consult your doctor or clinical research coordinator in advance.

[Prohibited concomitant drugs and therapies during Step 1 and Step 2]

· Antidepressants other than the study medicines and antipsychotics (including sulpiride)

· Mood stabilizers (Lithium carbonate, carbamazepine, valproic acid, lamotrigine, gabapentin, and other antiepileptic drugs) and St. John’s wort

· Specific psychotherapy for the treatment of depression, such as cognitive behavior therapy, electroconvulsive therapy, and repetitive transcranial magnetic stimulation.

4. Number of participants in the study and the examination period

In this study, we plan to recruit 242 patients, and the examination period is 52 weeks.

5. Advantages and disadvantages of participating in the study

1) Advantages

 Among all SSRI, escitalopram is a relatively new antidepressant drug that was released in 2011. It enhances serotonin activity in the central nervous system by inhibiting the reuptake of serotonin. Duloxetine, which is a representative SNRI, was released in 2010, and previous studies demonstrated that SNRI are more effective than SSRI.

We consider the results of the present study, by collating with previous findings, to provide important insights into treatment selection frequently encountered in clinical settings.

2) Disadvantages

**Escitalopram**

In domestic clinical trials (4 trials) targeting patients with MDD, side effects, including abnormal laboratory test values, were observed in 409 (74.4%) out of 550 cases. The main side effects noted were nausea (131 cases, 23.8%), somnolence (129 cases, 23.5%), headaches (56 cases, 10.2%), dry mouth (53 cases, 9.6%), floating dizziness (48 cases, 8.7%), malaise (39 cases, 7.1%), diarrhea (34 cases, 6.2%), and abdominal discomfort (32 cases, 5.8%) (at the time of approval).

[Severe side effects]

1) Convulsions (frequency unknown)

2) Syndrome of inappropriate secretion of antidiuretic hormone (SIADH) (frequency unknown)

SIADH accompanied by hyponatremia, headaches, lack of concentration, memory disturbance, confusion, hallucinations, convulsions, and syncope

3) Serotonin syndrome (frequency unknown)

Serotonin syndrome accompanied by anxiety, irritability, excitement, tremors, myoclonus, and hyperthermia

4) QT prolongation (frequency unknown), ventricular tachycardia (including torsades de pointes) (frequency unknown)

**Duloxetine**

In domestic clinical trials targeting patients with depression/a depressive state, side effects, including abnormal laboratory test values, were observed in 663 (90.2%) out of 735 cases. The main side effects noted were nausea (269 cases, 36.6%), somnolence (228 cases, 31.0%), dry mouth (168 cases, 22.9%), headaches (154 cases, 21.0%), constipation (102 cases, 13.9%), diarrhea (87 cases, 11.8%), dizziness (80 cases, 10.9%), elevated triglyceride levels (56 cases, 7.6%), abdominal pain (52 cases, 7.1%), elevated ALT levels (51 cases, 6.9%), insomnia (50 cases, 6.8%), malaise (45 cases, 6.1%), elevated AST levels (38 cases, 5.2%), and appetite reductions (38 cases, 5.2%) (at the time of approval).

[Severe side effects]

1) Serotonin syndrome (frequency unknown)

Serotonin syndrome accompanied by anxiety, irritability, excitement, tremors, myoclonus, and hyperthermia

2) SIADH (frequency unknown)

SIADH accompanied by hyponatremia, headaches, lack of concentration, memory disturbance, confusion, hallucinations, convulsions, and syncope

3) Convulsions (0.16%), hallucinations (frequency unknown)

4) Liver dysfunction, hepatitis, and jaundice (frequency unknown)

5) Skin mucosa ocular syndrome (Stevens-Johnson syndrome) (frequency unknown)

6) Anaphylactic reaction (frequency unknown)

Anaphylactic reaction accompanied by dyspnea, convulsions, angioedema, and urticaria

7) Hypertension crisis (frequency unknown)

Hypertension crisis accompanied by damage to organs such as the brain, heart, and kidneys due to a sudden increase in blood pressure

8) Urinary retention (frequency unknown)

Urinary retention is a state in which urine cannot be discharged even though there is urinary urgency.

6. Study discontinuation

➀ If there is an offer to cancel from you.

➁ If your doctor decides that it is difficult to continue this research because of your condition.

➂ If it becomes clear that your condition does not meet the criteria for participation in the study.

➃ When your doctor decides that it is necessary to use concomitant drugs that are prohibited according to your condition.

➄ If you were found to be pregnant during this study (in the case of a woman)

➅ When you become unable to visit the hospital due to moving or a hospital change.

➆ When the investigator’s doctor decides that it is necessary to stop the study.

When you discontinue the study, we may conduct inspections to assure your safety.

7. Other treatment options

 Various methods, including environmental control, such as taking a rest, medication, and psychotherapies, including cognitive behavior therapy, are currently used to treat depression based on the symptoms of and reasons for the disease.

1) Antidepressants

Antidepressant drugs other than SSRI and SNRI include tricyclic antidepressants and noradrenergic and specific serotonergic antidepressants (NaSSA), which improve symptoms by inhibiting the reuptake and modulating neurotransmitters related to cellular activity. Tricyclic antidepressants are known to have side effects such as dry mouth, constipation, orthostatic hypotension, urinary retention, and drowsiness, while NaSSA may cause fatigue, dizziness, and constipation.

2) Electroconvulsive therapy

3) Psychotherapy

Psychotherapy is defined as "a way to improve the condition through interactions between the therapist and patient".  Cognitive behavior therapy, one of the main approaches, is a means to assist patients to reconsider cognitive schema (how to view things), solve the emotional issue, and improve subjective awareness.

8. Protection of privacy (protection of personal information)

 In order to investigate whether the research is conducted properly, authorized persons (permitted to access the information), members of the Institutional Review Board (IRB), and persons of regulatory authorities may view your medical records and examination outcomes. However, the stakeholders are obligated to preserve confidentiality; therefore, your private information, such as your name and address, will never be leaked to other parties. By signing the research participation consent form, you have granted your approval.

9. Attribution of intellectual property rights arising from the research

 Intellectual property rights arising from the research will not occur.

10. Cost burden

Since this study is conducted during usual medical treatment, you must pay the medical cost using the health insurance system you are subscribed to.

As a compensation fee, we will provide you with a 3000 yen prepaid card (Quo card) three times during the examination period (at the baseline, at the end of the Step 2 treatment or discontinuation time, and at the end of the 52-week follow-up).

11. Disclosure of the research plan and personal information

We will disclose the research plan if you wish. In addition, we will inform you about your individual results.

12. Publication of research outcomes

The outcomes of this study and test results may be reported in scientific conferences and medical journals, and in this case, we assure you that your personal information, such as your name, will never be disclosed.

1 3. Funding source of the research

The funding source of this study is a research expenses contract from the Foundation of Neurology and Psychiatry Science Foundation, which was donated from Mochida Pharmaceutical Co., Ltd., Mitsubishi Tanabe Pharma Corporation, and Shionogi Pharmaceutical Co., Ltd.. During the study, if other new companies agree with the purpose of this clinical study and decided to donate to this foundation in order to support it, we will inform you.

1 4. Compensation for health damage

 In the case of health hazards during the protocol treatment (Step 1 and Step 2) and follow-up observation period of this study, your doctor will do his/her best to perform appropriate treatment. The treatment for health damage will be covered by your health insurance as well as usual medical treatment. In the case of severe adverse events due to the study medicine at the discretion of your doctor during the protocol treatment period (Step 1 and Step 2), compensation will be provided based on clinical trial insurance. However, if health damage is not related to this trial or is caused by your intentional or serious negligence, such as not following the instructions of your doctor, you may not be eligible for compensation.

This compensation system does not interfere with your right to claim damages (even if you sign the consent form, you may file a suit for damages if you find liability). If you have any questions about compensation, please contact your doctor.

1 5. Important points to comply with during the study period

➀ Please contact your doctor or clinical research coordinator whenever you cannot visit on the scheduled date.

➁ Take the study medicine as instructed by your doctor and inform us about medication adherence at the next visit.

➂ If you are taking any medication (i.e. over-the-counter drugs) or want to use new medicine after participating in the trial, please report it to your doctor or clinical research coordinator. There are some medicines that are not recommended for use during the trial.

➃ If you feel your physical condition is bad, please contact your doctor any time.

16. About the IRB

This study was investigated and approved by the "National Center of Neurology and Psychiatry Institutional Review Board". If you have any inquiries about the procedures, membership list, and deliberation details, please contact your doctor or clinical research coordinator.

« IRB »

Name of the clinical trial review committee:

National Center of Neurology and Psychiatry Institutional Review Board

Name of establisher of clinical trial judging committee:

National Center of Neurology and Psychiatry President

Location of the IRB: 4-1-1 Ogawa-Higashi, Kodaira, Tokyo, Japan.

About viewing documentation:

If you wish, you may view IRB procedures, membership lists, and deliberation summaries. If you want to view them, please contact your doctor or clinical research coordinator. In addition, these materials are posted on the following website.

http://www.ncnp.go.jp/committee/ethics.html

1 7. About inquiries

If you have any questions about this study, information you want to obtain, or any concerns, please feel free to ask your doctor or clinical research coordinator at any time without hesitation.

"Contact information"

National Center of Neurology and Psychiatry

〒 187 - 8551

4-1-1 Ogawa-Higashi, Kodaira, Tokyo

Tel: 042 (341) 2712 dial in (8:30 to 17:15)

Principle Investigator Kazuyuki Nakagome

Your doctor

In the case of an emergency, please contact the doctor in charge.

At night, on holidays (Saturday and Sunday), or public holidays, please call 042-341-2710 and tell the operator to be connected to the "psychiatrist on duty doctor". In this case, please tell the doctor that you are participating in "clinical research on antidepressants (ACCEPT study) ".

In the explanation provided herein, if you have anything you do not understand and need a more detailed explanation, please feel free to ask. Furthermore, please understand the contents of this study well and decide to participate after thoroughly studying it. If you decide to participate in this research, please sign the consent form. Please keep this explanation document and consent form.

**Consent Form: Long-term acceptability study on novel antidepressants for patients with major depressive disorder**

Dear President of the National Center of Neurology and Psychiatry,

I have been thoroughly informed about the study “**Long-term administration test of new antidepressants that target the major depressive disorder patients”** with the informed consent document and understood the following:

□ The purpose of the research

□ Voluntariness and freedom of withdrawal from research participation

□ The method of research

□ Advantages and disadvantages of participating in research

□ Privacy protection

□ Usage of research results and attribution of intellectual property rights arising from research

□ Costs or burden for participating

□ Disclosure of the research plan and personal information

□ Publication of research results

□ Funding sources for research

□ Compensation for health damage

I will participate in this research.

Date of agreement

Signature (authentic)

 I,  , explained this study based on the informed consent document, answered all questions, and obtained agreement from this participant.

Explanation date

Doctor’s signature

Research coordinator’s signature

Department of

National Center of Neurology and Psychiatry,

First Edition

Creation Date: October 10, 2013
